# *Her2/EGFR-PDGFR* pathway aberrations associated with tamoxifen response in metastatic breast cancer patients

**DOI:** 10.1186/s43046-022-00132-5

**Published:** 2022-07-25

**Authors:** Ibrahim Malash, Osman Mansour, Rabab Gaafar, Sabry Shaarawy, Mona S. Abdellateif, Ola S. Ahmed, Abdel-Rahman N. Zekri, Abeer Bahnassy

**Affiliations:** 1 Medical Oncology Department, National Cancer Institute (NCI), Cairo University, Cairo, 11976 Egypt; 2 Medical Biochemistry and molecular biology, Cancer Biology Department, NCI, Cairo University, Cairo, 11976 Egypt; 3 Molecular Virology and Immunology Unit, Cancer Biology Department, NCI, Cairo University, Cairo, 11976 Egypt; 4 Tissue culture and Cytogenetics Unit, Pathology Department, NCI, Cairo University, 1 Fom El- Khalig street, Cairo, 11976 Egypt

**Keywords:** Breast, Cancer, Tamoxifen, *EGFR*, *JAK1*, *COL1A1*, *GAB1*, *FN1*, *MKNK1*

## Abstract

**Background:**

Metastatic breast cancer (MBC) is a major health problem worldwide. Some patients improve on tamoxifen and others do not respond to treatment. Therefore, the aim of the current study is to assess genetic aberrations in the *Her2/EGFR-PDGFR* pathway associated with tamoxifen response in MBC patients.

**Methods:**

This is a retrospective cohort study, including 157 hormone receptors positive, locally recurrent inoperable and/or MBC patients on tamoxifen treatment. Patients were categorized into 78 (49.7%) tamoxifen responders and 79 (50.3%) tamoxifen non-responder patients. Genetic aberrations of 84 genes involved in the *Her2/EGFR-PDGFR* pathway were assessed in the tumor tissue samples obtained from the patients using SA-Bioscience assay. The identified panel was correlated to patients’ response to treatment, to detect the differentially expressed genes in tamoxifen responders and non-responders.

**Results:**

One hundred twenty-three (78.3%) patients were estrogen receptor (ER) and progesterone receptor (PR) positive, 108 (68.8%) were ER only positive, and 78 (49.7%) were PR only positive. There were 56 genes overexpressed in the refractory group compared to responders. However, only five out of these 56 genes, Janus kinase 1 *(JAK1)*, collagen type I alpha 1 *(COL1A1)*, *GRB2*-associated binding protein 1 *(GAB1)*, fibronectin-1 *(FN1)*, and *MAP* kinase-interacting serine/threonine-protein kinase (*MKNK1)*, showed statistical significance between the two groups. Patients with bone metastasis showed a better response to treatment compared to those with metastatic deposits in other sites such as visceral metastasis (*P* < 0.005).

**Conclusions:**

Genetic profiling using simple quantitative real-time polymerase chain reaction (qRT-PCR) protocols could be used to assess response to tamoxifen treatment in MBC patients. According to our data, a five-gene panel in the *EGFR* pathway (*JAK1*, *COL1A1*, *GAB1*, *FN1* and *MKNK1*) could be used to categorize MBC patients into groups according to treatment response.

## Background

Breast cancer (BC) is the most commonly diagnosed cancer in females, and it is the leading cause of cancer-related death among women worldwide [1]. It is a heterogeneous disease with varied response to treatment and survival rates. In Egypt, BC represented ~ 38.2% of all female malignancies [2]. Patients with MBC often have a bad prognosis, poor response to treatment, and reduced survival rates. Thus, accurate diagnostic and prognostic biomarkers for such patients are highly needed to achieve proper management, as well as to improve disease outcomes and survival rates. The majority of BC patients (> 60%) usually express ER and/or PR, and those patients are considered proper candidates for hormonal therapy that blocks estrogen signaling in BC cells [3]. Tamoxifen which is an effective hormonal therapy and a selective estrogen receptor modulator (SERM) is used for early as well as advanced ER/PR+ BC patients. It has been shown that tamoxifen treatment significantly improves both relapse-free and overall survival rates in those patients [4]. However, tamoxifen resistance occurs in about third of the patients, which finally leads to disease progression and metastasis [5, 6]

Several mechanisms have been reported to explain the acquired resistance to tamoxifen, such as activation of some genetic pathways including *EGFR/HER-2* pathways, *RAS/RAF/MEK/MAPK* signaling pathway, *PI3K/AKT*, and *mTOR* or *PTEN* signaling pathways [7, 8]. Accordingly, gene expression analysis and molecular profiling are highly required to define gene signatures that can accurately predict clinical responses and outcome of the breast cancer patients, in addition to identify new therapeutic options for those who develop tamoxifen resistance [9].

Therefore, the aim of the current study is to assess genetic aberrations (upregulations and/or downregulations) in a specific panel of genes involved in *EGFR/PDGFR* pathway in MBC patients*.* These genetic aberrations will be correlated to the patients’ response to tamoxifen treatment. We think that the emerged array data could permit identifying potential target panels of genes that could be used for early detection, diagnosis, prognosis, and prediction of response to treatment in those patients, as well as for the proper choice of therapy. These panels may also alleviate the drug resistance and improve the clinical outcome of MBC patients.

## Methods

### Patients

This is a prospective cohort study which included 157 MBC patients who were diagnosed and treated at the National Cancer Institute during the period from January 2018 to December 2019. The selection criteria of the patients were those who presented with metastatic or locally recurrent inoperable disease, and none of them received prior hormonal therapy (tamoxifen). Patients with rapidly progressive and/or large volume visceral metastases, central nervous system (CNS) metastases, or inflammatory breast cancer were excluded before enrollment in the study. Similarly, patients with any contra-indication to tamoxifen and those with prior deep venous thrombosis and/or anti-coagulant medication within 2 weeks of registration were also excluded. Concomitant pain medications were freely allowed, as well as local palliative radiotherapy for painful or high-risk bone metastases. Bisphosphonates were also freely allowed in cases with bone involvement whenever indicated.

All patients were subjected to full history taking and clinical examination, as well as routine biochemistry and hematological tests. Patients were also evaluated for tumor response after 4, 8, 16, and 24 weeks of treatment, and they were categorized into tamoxifen responders, those who achieved and maintained good clinical response to tamoxifen for more than 6 months, and tamoxifen resistant, those who progressed during the first 6 months of treatment.

### Sample preparation and RNA extraction

Total ribonucleic acid (RNA) was extracted from the formalin-fixed paraffin-embedded tissue samples (FFPETs) of breast cancer patients, using commercially available kits according to the manufacturer’s protocols (RNeasy Mini Kit, Qiagen, Milan, Italy). The extracted RNA was reverse transcribed using RT2 First Strand Kit (QIAGEN, USA) according to the manufacturer’s instructions.

### Gene profiling array

The expression levels of 84 genes in the *Her2/EGFR-PDGFR* pathway were assessed using the SA-Bioscience real-time array (QIAGEN kits; Patch or Cat. No PAXX-040Y). The array process was performed with 25 μl RT2 SYBR Green qPCR Master mix in each well of the PCR Array plate that containing the gene-specific primer sets. The analysis was done using AB-Applied Biosystem, 7500 Fast PCR.

The expression levels of all genes were assessed in responders (the control group) and the refractory patients (tested group). Then, the data regarding patients’ profiles were correlated to the relevant clinicopathological features of the patients and their response to hormonal therapy (Table 2).

### Statistical analysis

The sample size was calculated using the online power test for sample size calculation, taking into consideration the incidence of the primary outcome in the population and the study group with an α-error of 0.05 and power of 80%. The data of the patients were analyzed using SPSS version 24 (IBM SPSS, Armonk, NY, USA). Qualitative data were described as frequencies and percentages. The relation between qualitative data was determined using the chi-square test or the non- parametric Fisher’s exact test as appropriate. Probability (*P* value) equal or less than 0.05 was considered statistically significant.

## Results

### Clinicopathological features of the patients

The mean age of the patients was 51.63 ± 10.5 years, and the median was 52 years. Eighty-one patients (51.6%) were > 50 years, and 76 (48.4%) were < 50 years. The minimum age of the patients was 29 years, and the maximum was 79. Thirty-one patients (19.7% of all tested cases) had a history of previous hormonal contraceptive pills intake, 73 patients (46.5%) were premenopausal, and 84 (53.5%) were postmenopausal. All patients presented with The Eastern Cooperative Oncology Group (ECOG) performance status ≤ 2, and they were almost equally distributed between the 2 studied groups (Table 1).

**Table 1:** Relevant clinicopathological features of all studied breast cancer patients

Characteristics	***N*** = 157 (%)
**Age**	
Mean: 51.63 ± 10.5	
< 50	76 (48.4)
≥ 50	81 (51.6)
**Tumor size**	
≤ 5 cm	131 (83.4)
> 5 cm	26 (16.6)
**TNM staging-T**	
T1	13 (8.3)
T2	104 (66.2)
T3	40 (25.5)
**Lymph nodes**	
N0	30 (19.1)
N1	51 (32.5)
N2	51 (32.5)
N3	25 (15.9)
**Grade**	
1	11(7)
2	112 (71.3)
3	34 (21.65)
**ER**	
Negative	49 (31.2)
Positive	108 (68.8)
**PR**	
Negative	79 (50.3)
Positive	78 (49.7)
**HER2**	
Negative	99 (63.1)
Positive	58 (36.9)

### Response to tamoxifen treatment

Among the studied cohort (157 patients), 78 patients (49.7%) responded to tamoxifen treatment, while 79 patients (50.3%) did not respond to treatment (refractory group). Our data showed a significant association between patient’s response to tamoxifen and the site of metastasis, as patients with visceral metastasis showed poor response to treatment compared to those with bone metastasis (*P* = 0.005). This significance was independent of other prognostic factors including the age of the patients and/or *Her-2neu* gene status, which were equally distributed between the two studied groups. There was no significant association between response to tamoxifen treatment and other clinicopathological features of the patients including age, menstrual status, contraceptive pills intake, tumor size, or histological grade (Table 2).

**Table 2:** Patients’ characteristics and their relation to tamoxifen response

Feature	Responders (78)	Refractory (79)	*P* value
Age (years)			
> 50 (81)	40 (51.3%)	41 (51.9%)	1.0
≤ 50 (76)	38 (48.7%)	38 (48.1%)	
Menstrual status			
Pre (73)	36 (46.2%)	37 (46.8%)	1.0
Post (84)	42 (53.8%)	42 (53.2%)	
Histological grade			
II (114)	57 (73.1%)	57 (72.2%)	0.69
III (43)	21 (26.9)	22 (27.8%)	
Contraception			
Yes (31)	17 (21.8%)	14 (17.7%)	0.55
No (126)	61 (78.2%)	65 (82.3%)	
Her-2 status			
+ve (58)	31 (39.7%)	27 (34.2%)	0.51
−ve (99)	47 (60.3%)	52 (65.8%)	
ER			
+ve (108)	52 (66.7%)	56 (70.9%)	0.61
−ve (49)	26 (33.3%)	23 (29.1%)	
PR			
+ve (78)	38 (48.7%)	40 (50.6%)	1.00
−ve (79)	40 (51.3%)	39 (49.4%)	
Disease site			
Bone only (46)	31 (39.7%)	15 (18.9%)	0.005*
Visceral (111)	47 (60.3%)	64 (81.1%)	

### Array profiling

**Fig. 1:**
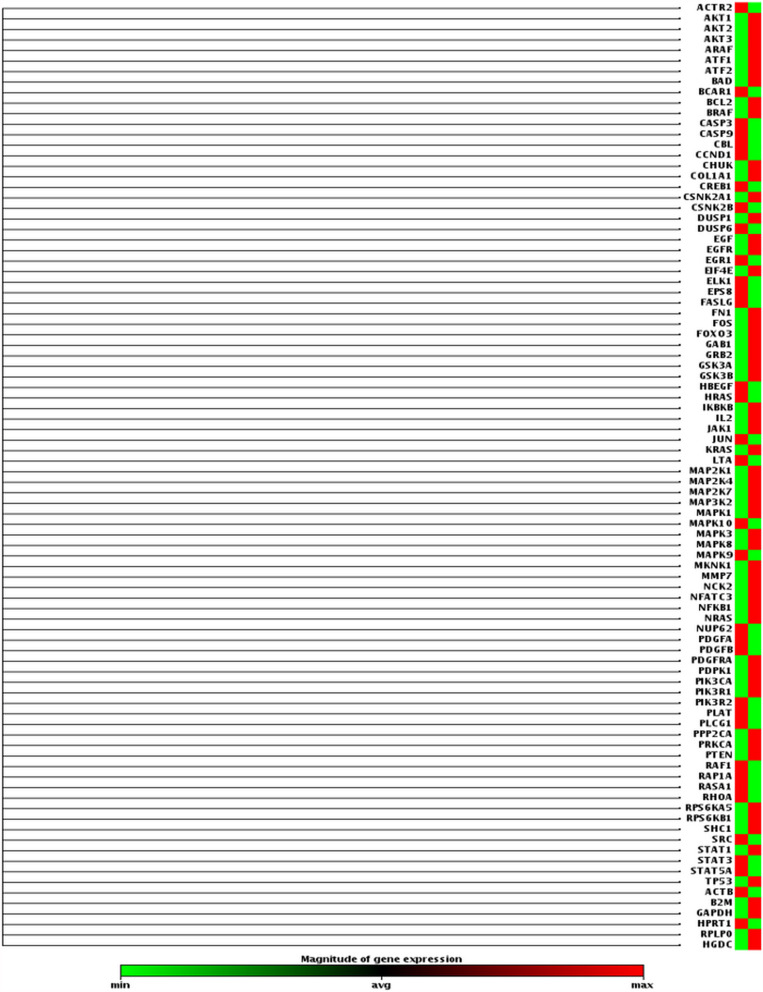
Array profiling of EGFR gene expression in breast cancer patients

The expression levels of 84 genes in the *Her2/EGFR-PDGFR* pathway were assessed using the SABioscience real time array system (Fig. 1). The array showed that 56 genes were differentially overexpressed in the refractory group compared to the responders. Those genes are as follows: *AKT1*, *AKT2*, *AKT3*, *ARAF*, *ATF1*, *ATF2*, *BAD*, *BCL2*, *BRAF*, *CHUK*, *COL1A1*, *CSNK2A1*, *DUSP1*, *EGF*, *EGFR*, *EIF4E*, *FN1*, *FOS*, *FOXO3*, *GAB1*, *GRB2*, *GSK3A*, *GSK3B*, *IKBKB*, *IL2*, *JAK1*, *KRAS*, *MAP2K1*, *MAP2K4*, *MAP2K7*, *MAP3K2*, *MAPK1*, *MAPK3*, *MAPK8*, *MKNK1*, *MMP7*, *NCK2*, *NFATC3*, *NFKB1*, *NRAS*, *PDGFRA*, *PDPK1*, *PIK3CA*, *PIK3R1*, *PPP2CA*, *PRKCA*, *PTEN*, *RPS6KA5*, *RPS6KB1*, *SHC1*, *STAT1*, *TP53*, *B2M*, *GAPDH*, *RPLP0*, and *HGDC.* Among those 56 genes, only 4 reached a statistically significant level: *JAK1* (*P* < 0.001), *FN1* (*P* < 0.02), *GAB1 (P* < 0.04), and *MKNK1 (P*< 0.05) and one gene *(COL1A1)* showed border line significance (*P* = 0.06, Table 3).

**Table 3:** The differentially overexpressed genes and their significance in refractory breast cancer patients

No.	Genes	***P*** value	No.	Genes	***P*** value
1	AKT1	0.30	29	MAP2K4	0.76
2	AKT2	0.78	30	MAP2K7	0.28
3	AKT3	0.18	31	MAP3K2	0.22
4	ARAF	0.98	32	MAPK1	0.26
5	ATF1	0.25	33	MAPK3	0.15
6	ATF2	0.56	34	MAPK8	0.54
7	BAD	0.86	35	***MKNK1***	***0.05***
8	BCL2	0.80	36	MMP7	0.67
9	BRAF	0.37	37	NCK2	0.18
10	CHUK	0.50	38	NFATC3	0.71
11	**COL1A1**	**0.06**	39	NFKB1	0.36
12	CSNK2A1	0.57	40	NRAS	0.31
13	DUSP1	0.32	41	PDGFRA	0.35
14	EGF	0.35	42	PDPK1	0.43
15	EGFR	0.15	43	PIK3CA	0.79
16	EIF4E	0.61	44	PIK3R1	0.30
17	**FN1**	**0.02**	45	PPP2CA	0.34
18	FOS	0.43	46	PRKCA	0.66
19	FOXO3	0.37	47	PTEN	0.34
20	**GAB1**	**0.04**	48	RPS6KA5	0.43
21	GRB2	0.26	49	RPS6KB1	0.13
22	GSK3A	0.38	50	SHC1	0.16
23	GSK3B	0.36	51	STAT1	0.53
24	IKBKB	0.82	52	TP53	0.21
25	IL2	0.62	53	B2M	0.66
26	**JAK1**	**0.001**	54	GAPDH	0.90
27	KRAS	0.53	55	RPLP0	0.38
28	MAP2K1	0.75	56	HGDC	0.12

**Fig. 2:**
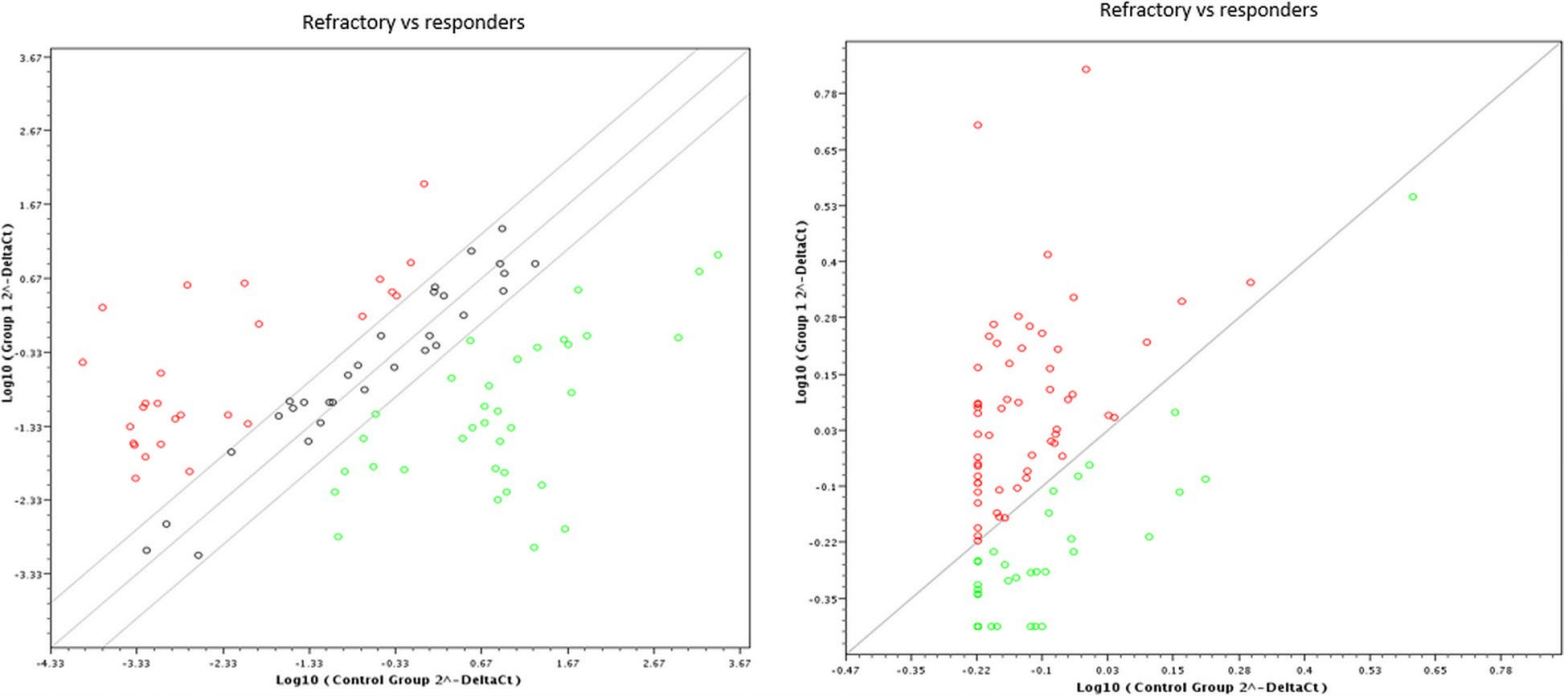
The array analysis of the studied genes in the EGFR pathway showing the differential expression of the tested genes. The red circles represent the overexpressed genes the black circles represent normally expressed genes, and the green circles represent genes with reduced expression

**Fig. 3:**
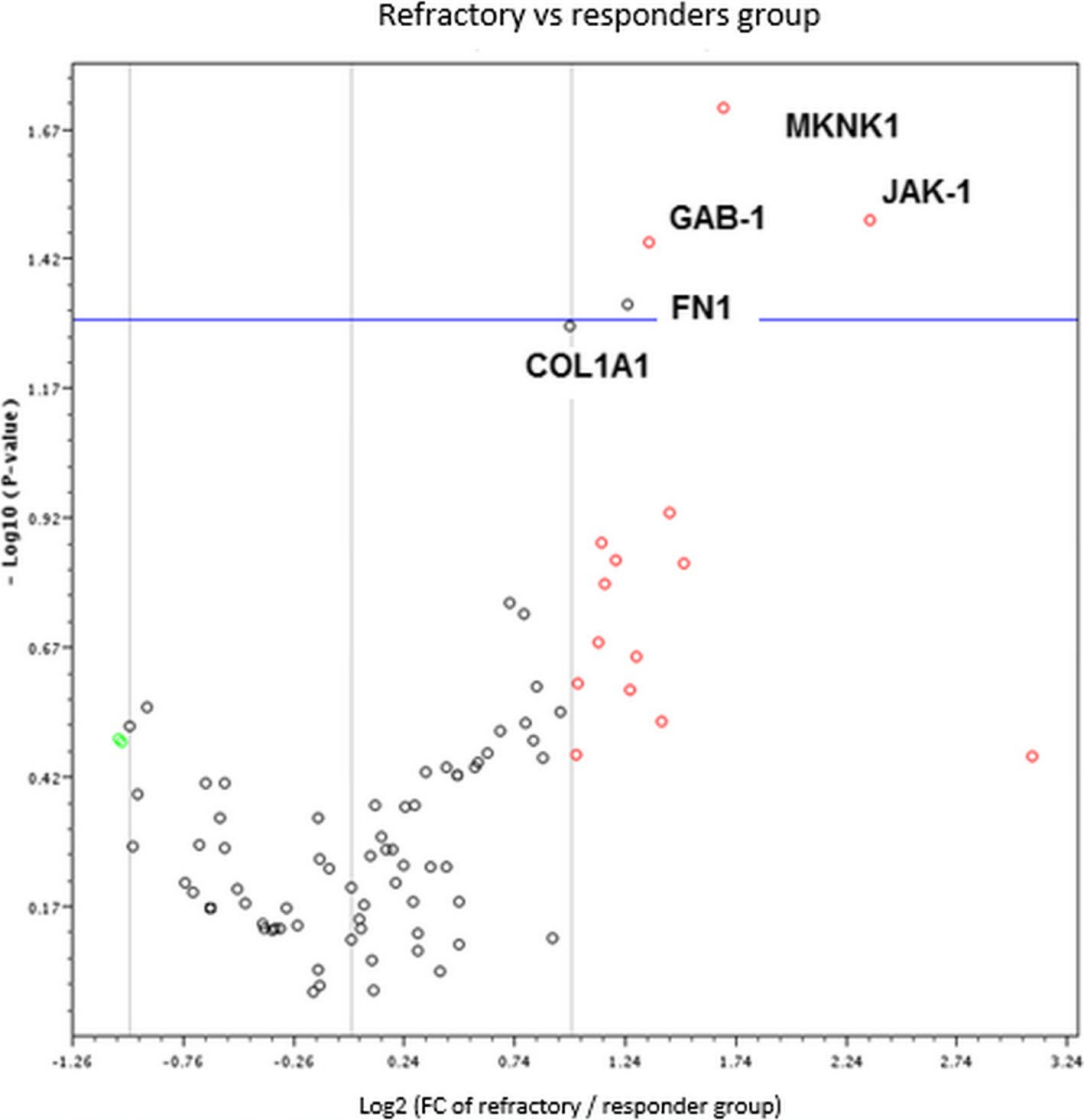
The volcano plot for the tested genes in the EGFR pathway. The upper right square shows the 4 genes that were significantly expressed between the responders and the refractory groups

On the other hand, 34 genes were differentially underexpressed (reduced expression) in the refractory group compared to responders (control group). Those are *ACTR2*, *BCAR1*, *CASP3*, *CASP9*, *CBL*, *CCND1*, *CREB1*, *CSNK2B*, *DUSP6*, *EGR1*, *ELK1*, *EPS8*, *FASLG*, *HBEGF*, *HRAS*, *JUN LTA*, *MAPK10*, *MAPK9*, *NUP62*, *PDGFA*, *PDGFB*, *PIK3R2*, *PLAT*, *PLCG1*, *RAF1*, *RAP1A*, *RASA1*, *RHOA*, *SRC*, *STAT3*, *STAT5A*, *ACTB*, and *HPRT1.* However, none of these genes reached a statistically significant level (Figs. 2 and 3 and Table 4).

**Table 4:** The differentially underexpressed genes and their significance in refractory breast cancer patients

No.	Genes	***P*** value	No.	Genes	***P*** value
1	ACTR2	0.33	18	MAPK10	0.68
2	BCAR1	0.68	19	MAPK9	0.75
3	CASP3	0.39	20	NUP62	0.74
4	CASP9	0.96	21	PDGFA	0.55
5	CBL	0.39	22	PDGFB	0.75
6	CCND1	0.64	23	PIK3R2	0.73
7	CREB1	0.57	24	PLAT	0.68
8	CSNK2B	0.99	25	PLCG1	0.52
9	DUSP6	0.68	26	RAF1	0.68
10	EGR1	0.30	27	RAP1A	0.52
11	ELK1	0.67	28	RASA1	0.61
12	EPS8	0.39	29	RHOA	0.32
13	FASLG	0.90	30	SRC	0.46
14	HBEGF	0.68	31	STAT3	0.28
15	HRAS	0.75	32	STAT5A	0.46
16	JUN	0.41	33	ACTB	0.75
17	LTA	0.62	34	HPRT1	0.52

## Discussion

In the current study, we tried to clarify the molecular mechanism(s) which might be involved in the development of resistance to treatment in metastatic breast cancer patients from Egypt. The tested patients were properly selected as follows: (1) all patients enrolled in the study were assessed according to the international guidelines and NCI protocols and (2) all required data regarding the tumor characteristics, treatment protocols, survival rates, and follow-up data were present in details in patients’ files. To achieve this goal, we assessed the molecular profiles of the tested patients using the *Her2/EGFR-PDGFR* pathway, which is commonly involved in metastatic and non-metastatic breast cancer. We sought that this might help in identifying specific gene panels (signatures) that can accurately predict patient’s response to endocrine therapy (tailored or targeted therapy). In addition, finding novel treatment targets in such patients group.

The current study demonstrated that patients with bone-only metastasis showed good and maintained response to tamoxifen (regardless of other studied factors), compared to those with visceral involvement. Based on this data, patients with bone only disease will get benefit from tamoxifen administration compared to those with visceral metastasis.

According to our results, 58 patients (36.94%) showed Her-2 gene amplification. This percentage is much higher than most reported data in the literature which show *Her-2* gene amplification in 15 to 30% of the patients assessed [10, 11] only. One possible explanation is that all patients enrolled in the study were hormone receptor positive. Furthermore, our data revealed no significant association between *Her-2* over expression and resistance to tamoxifen, since resistance in patients who expressed *Her-2* amplification was almost the same as in those who did not show *Her-2 neu* gene amplification.

Genetic profiling of the *EGFR* pathway using the (84) genes super array showed differential expression of some tumor suppressor genes including *TP53* and *PTEN*, the *ACT*, *RAF*, *BAD*, *Bcl-2*, *EGFR*, *JAK*, *RAS*, *MAPK*, *PDGFR*, *STATs*, and *SRC*, as well as other genes in this cascade.

Our data from Egypt showed that 56 genes were overexpressed in the refractory patients; however, only four genes reached a statistically significant level. The first gene is *MKNK1*, which is the gene coding for an enzyme (*MAP* kinase-interacting serine/threonine-protein kinase). This gene has an important role in tumorigenesis and tumor progression [12–14]. It is usually differentially overexpressed in breast cancer patients as previously mentioned by García-Recio et al. [15], who found that the *MKNK1* is usually overexpressed in several types of solid tumors among which are the breast and colorectal carcinomas, compared to their expression in the non-neoplastic and the normal tissues. In addition, Geter et al. [16] demonstrated that tamoxifen-resistant in ER+ breast cancer specimens showed increased *MNK* phosphorylation of *eIF4E*, which promotes tamoxifen resistance through selective mRNA translational reprogramming. Moreover, many recent studies have also suggested the possibility of targeting *MNK* kinases in solid tumors that overexpressing this protein [17, 18].

Another gene that was overexpressed in our assessed breast cancer patients is the *GAB1.* Its overexpression has been observed in several human cancers including breast and lung carcinomas [19]. In agreement with our data, Veeraraghavan et al. [20] reported that *ESR1-CCDC170* rearrangement induces hormonal treatment resistance in estrogen receptor-positive breast cancer through engaging the *GAB1* signalosome. Moreover, it had been reported in recent studies that *GAB1* has an important role in breast cancer aggressiveness, as it promotes tumor metastasis and progression [21, 22].

Our tested panel also identified *FN1* and *COL1A1* genes, which encode for the fibronectin-1 and collagen type I alpha 1, respectively. They play major role(s) in cell adhesion and migration processes [23]. The present results demonstrated their differential overexpression in refractory breast cancer patients, though revealing a borderline significance (*P* = 0.06). These results are in line with Wang et al. [24] and Nolan et al. [25], who reported that *COL1A1* and *FN1* could be considered as cancer stromal key genes associated with breast cancer invasion and metastasis. They concluded that over expression of these genes associated significantly with an advanced stage of breast cancer patients as well as poor clinical outcome. Similarly, in an interesting study done by Mucaki et al. [26], they were able to identify a panel of 26-gene signature, among them the *FN1* gene. This panel can accurately predict patients’ survival and outcome after paclitaxel therapy. Our data regarding *COL1A1* expression are consistent with the previously reported data of Xiong et al. [27], who provided evidence that *Col1A1*, *Col3A1*, and *Col4A1* are overexpressed during breast cancer development and progression. Similarly, Akkiprik et al. [28] showed that *Col1A1* gene is upregulated in the group of breast cancer patients compared to the control group (normal breast tissues). However, none of the previously mentioned studies correlate the expression of *COL1A1* and *FN1* with tamoxifen treatment.

Moreover, the *JAK1*, as well as the signal transducer and activator of transcription (*STAT)* genes signaling pathways proved to be involved in cell growth, differentiation, and immune function [29]. It was also proved that genetic variations in those genes are highly related to breast cancer-specific mortality [27]. The current study showed that *JAK1* gene was significantly overexpressed in tamoxifen resistant breast cancer patients, compared to the responder group. These data are consistent with the recently published study by Chen et al. [30], who used IMPALA (Inferred Modularization of PAthway LAndscapes) integrated information from high throughput gene expression experiments and genome-scale knowledge databases to identify aberrant pathways associated with breast cancer progression. They reported that *JAK-STAT* signaling pathway associated significantly with breast cancer recurrence and tamoxifen resistance in ER+ breast cancer patients.

Our results show that many other genes (34 genes) in the tested array were down regulated in the refractory group; however, none of these genes reached a statistically significant level.

The limitations in the present study were that the patients were recruited from a single institute with a limited number of patients. Therefore, these data should be validated on a larger number of patients recruited from different regions in Egypt. Also, the identified panel of genes (*JAK1*, *COL1A1*, *GAB1*, *FN1*, and *MKNK1*) should be studied extensively and correlated to the other clinic-pathological features of the BC patients including mainly the patients’ survival rates and outcomes.

## Conclusions

We concluded that the identified five panel genes in the *Her2/EGFR-PDGFR* pathway (*JAK1*, *COL1A1*, *GAB1*, *FN1*, and *MKNK1*) could be used to predict response to tamoxifen in metastatic BC patients from Egypt. This will make them potential target therapy for metastatic breast cancer, though this has to be verified in an extended study including larger samples.

## Data Availability

Data supporting the findings are included in the manuscript, and any additional data are available at the corresponding author on request.

## Abbreviations

CNS: Central nervous system
*COL1A1*: Collagen type I alpha 1
ER: Estrogen receptor
FFPET: Formalin fixed paraffin embedded tissue
*FN1*: Fibronectin-1
*GAB1*: *GRB2*-associated binding protein 1
IMPALA: Inferred Modularization of PAthway LAndscapes
*JAK1*: Janus kinase 1
MBC: Metastatic breast cancer
*MKNK1*: *MAP* kinase-interacting serine/threonine-protein kinase
PR: Progesterone receptor
SERM: Selective estrogen receptor modulator
*STAT*: Signal transducer and activator of transcription
